# Investigation of Degradation and Biocompatibility of Indirect 3D-Printed Bile Duct Stents

**DOI:** 10.3390/bioengineering11070731

**Published:** 2024-07-19

**Authors:** Ming-Chan Lee, Cheng-Tang Pan, Ruo-Jiun Huang, Hsin-You Ou, Chun-Yen Yu, Yow-Ling Shiue

**Affiliations:** 1 Department of Electrical Engineering, National Kaohsiung University of Science and Technology, Kaohsiung 807, Taiwan; mclee@nkust.edu.tw; 2 Department of Mechanical and Electro-Mechanical Engineering, National Sun Yat-sen University, Kaohsiung 804, Taiwan; pan@mem.nsysu.edu.tw (C.-T.P.); royhuang@mem.nsysu.edu.tw (R.-J.H.); 3 Institute of Advanced Semiconductor Packaging and Testing, College of Semiconductor and Advanced Technology Research, National Sun Yat-sen University, Kaohsiung 804, Taiwan; 4 Institute of Precision Medicine, National Sun Yat-sen University, Kaohsiung 804, Taiwan; 5 Taiwan Instrument Research Institute, National Applied Research Laboratories, Hsinchu City 300, Taiwan; 6 Liver Transplantation Program, Departments of Diagnostic Radiology and Surgery, Kaohsiung Chang Gung Memorial Hospital, Chang Gung University College of Medicine, Kaohsiung 833, Taiwan; ouhsinyou@gmail.com; 7 Institute of Biomedical Sciences, National Sun Yat-sen University, Kaohsiung 804, Taiwan

**Keywords:** bile duct stent, short-term treatment, indirect 3D printing, degradation, biocompatibility

## Abstract

This study proposes a bile duct stent based on indirect 3D printing technology. Four ratio materials were synthesized from lactic acid (LA) and glycolide (GA) monomers by melt polymerization: PLA, PLGA (70:30), PLGA (50:50), and PLGA (30:70). The four kinds of material powders were preliminarily degraded, and the appearance was observed with an optical microscope (OM) and a camera. The weight and appearance of the four materials changed significantly after four weeks of degradation, which met the conditions for materials to be degraded within 4–6 weeks. Among them, PLGA (50:50) lost the most—the weight dropped to 13.4%. A stent with an outer diameter of 10 mm and an inner diameter of 8 mm was successfully manufactured by indirect 3D printing technology, demonstrating the potential of our research. Then, the degradation experiment was carried out on a cylindrical stent with a diameter of 6 mm and a height of 3 mm. The weight loss of the sample was less than that of the powder degradation, and the weight loss of PLGA (50:50) was the largest—the weight dropped to 79.6%. The nano-indenter system measured the mechanical properties of materials. Finally, human liver cancer cells Hep-3B were used to conduct in vitro cytotoxicity tests on the scaffolds to test the biocompatibility of the materials. A bile duct stent meeting commercial size requirements has been developed, instilling confidence in the potential of our research for future medical applications.

## 1. Introduction

With an aging population and the increasing incidence of chronic diseases, the global medical device market continues to expand. The global biliary stent market size exceeded USD 320 million in 2021 and is expected to grow at a compound annual growth rate (CAGR) of 5.0% during 2022–2028 [1]. According to statistics from the Ministry of Health and Welfare of the Republic of China, the number of cancer deaths in 2010 ranked first among all causes of death in the country, and liver and intrahepatic bile duct cancer ranked second among the top ten causes of cancer death [2]. In the past ten years, the number of new patients with liver and intrahepatic bile ducts has also continued to increase. From 1999 to 2019, patients increased from 11,023 to 16,233 [3]. It can be seen that the treatment of bile duct obstruction cannot be ignored.

Clinical conditions requiring biliary stent implantation are divided into benign conditions and malignant tumors. Benign conditions: (1) For benign biliary strictures, multiple plastic bile duct stents or covered/partially covered metal stents are commonly used to provide smooth bile ducts, and the stents are replaced every three months. (2) If the bile leak does not cause lesions, a plastic biliary stent is inserted without sphincterotomy (EST), and the stent is removed after 4 to 6 weeks. (3) For refractory gallstones, if the stones cannot be removed after ERCP or endoscopic papillary balloon dilation (EPBD) treatment, a temporary (for example, three months) plastic stent is placed, combined with oral ursodeoxycholic acid, which reduces the risk of cholangitis due to stent placement. For malignant hilar obstruction and malignant non-hilar bile duct obstruction caused by malignant tumors, bile duct stents of different materials will be used according to the life expectancy: plastic bile duct stents will be used if the life expectancy is less than four months. Metal bile duct stents will be used if the life expectancy is over four months [4]. Stent therapy for biliary obstruction can be divided into three stages: placement, replacement or removal, and postoperative follow-up [5,6].

In recent years, humans have gradually developed biodegradable polymers. Polyest ers, polyamides, polyurethanes, polyanhydrides, synthetic polymers, and hydrolyzable leading chains can biodegrade under specific conditions. The degradation rate [7] can be controlled by selecting different materials or scaffold manufacturing methods. Currently, there are a few commercially available biodegradable biliary stents, such as DV STENT BILIARY (ELLA-CS, s.r.o., Hradec Kralove, Czech Republic), ARCHIMEDES™* (Medtronic, Watford, United Kingdom), and UNITY-B (amg Internationall, Winsen, Germany). The degradation rates of the stents are 3–6 months, 12/20/77 days, and 1–3/3–6/6+ months [8,9,10]. Common polymer scaffold manufacturing techniques include solvent casting and particle leaching, emulsion freeze-drying, and electrospinning 3D printing, among others [11]. The diameter of the human common bile duct ranges from 2 to 7.9 mm, and the overall average is 4.1 ± 1.01 mm [12]. Therefore, the bile duct stent is mainly in a round tube, which is more suitable for 3D printing technology. There have been many studies on implants based on fused deposition modeling (FDM) 3D printing technology: 3D-printed polycaprolactone (PCL) scaffolds embedded with PGA sutures are applied to bone substitutes [13], PCL drug-eluting stents added with graphene are used to treat coronary artery blockage [14], and FDM-based printing plus the freeze-drying/particle leaching method and polylactic acid/polycaprolactone/hydroxyapatite (PLA/PCL/HA) composite materials are used to make bone scaffolds, and so on [15,16].

Due to their unique properties and advantages over other bioresorbable materials, PLA and poly (lactic-co-glycolic acid) (PLGA) are selected for bioresorbable applications. These polymers are particularly favored in biomedical applications due to their biocompatibility, biodegradability, and adjustable degradation rates, which can be tailored to match the specific needs of various medical applications. One significant advantage of PLA and PLGA is their ability to degrade into non-toxic byproducts (lactic acid and glycolic acid) that are naturally metabolized by the body, thus minimizing the risk of chronic inflammation often associated with nonbioresorbable materials. This degradation process also allows for the gradual transfer of load to healing tissue, which is critical in applications such as tissue engineering scaffolds and drug delivery systems. Furthermore, the mechanical properties of PLA and PLGA can be precisely controlled through copolymer ratios and molecular weight adjustments, providing a range of materials from rigid to flexible to suit different applications. This adaptability is less readily achievable with other bioresorbable polymers, which often have fixed properties and degradation rates. These attributes make PLA and PLGA highly advantageous for applications requiring temporary support or delivery vehicles that safely integrate and disappear from the body, avoiding surgical removal and reducing patient trauma and recovery time.

These stents are made from PLA and PLGA synthesized from lactic acid (LA) and glycolic acid (GA) monomers. To meet clinical needs, the critical design specifications of the stents include the following:

Material composition: Selection of PLA and three different ratios of PLGA (70:30, 50:50, and 30:70) to achieve varying degradation rates and mechanical properties. The choice of PLGA (50:50) is critical due to having the fastest degradation rate, making it suitable for short-term applications.

Size specifications: The stent is designed with an outer diameter of 10 mm and an inner diameter of 8 mm to meet commercial standards and ensure appropriate support and fluid dynamics.

Manufacturing process: Utilization of indirect 3D printing technology, which allows precise control over the shape and structure of the stent, thus achieving the desired mechanical strength and degradation behavior.

Mechanical performance: Testing the material’s Young’s modulus and hardness using a nano-indentation system to ensure that the stent can withstand the necessary loads in vivo while maintaining structural integrity during degradation.

These design specifications aim to develop a bile duct stent that degrades within 4 to 6 weeks, is suitable for short-term therapeutic applications, and fully degrades in the body to avoid the need for secondary surgical removal.

No biodegradable biliary stent degrades within 4–6 weeks for patients with bile leaks. Since most polymers degrade for a long time [17,18,19,20,21,22,23], PLGA [24] materials can control degradation through different ratios of LA and GA, among which PLGA (50:50) showed the highest degradation rate. Currently, there are commercially available bile duct stents, such as the uncovered stent produced by Taewoong Niti-S™ Biliary D Stent (Taewoong Medical, Gyeonggi-do, South Korea), primarily made of nitinol with a diameter of 8–10 mm [25]. WallFlex™ Biliary RX Stents (Boston Scientific, Marlborough, MA, USA) produce covered stents with an outer layer of nitinol and an inner layer of platinum, with diameters of 8–10 mm [26]. The Cotton-Leung^®^ Biliary Stent (Cook Medical, Bloomington, IN, USA) produces plastic bile duct stents made of polyethylene, with diameters ranging from 5 to 11.5 mm [27]. Commercially available bile duct stents are mainly categorized into plastic and metal bile duct stents. The materials of plastic bile duct stents primarily include polyethylene (PE), polyurethane (PU), and Teflon, with various shapes to help stabilize the stent and prevent displacement [28]. Metal bile duct stents are composed of metal alloys such as stainless steel and nitinol. Their diameter is approximately 6–10 mm. Although metal stents have a more vital radial force than plastic stents, excessive pressure can cause bile duct damage or inward growth of bile duct mucosal tissue, making them difficult to remove. The most common diameters for commercially available bile duct stents, including plastic and metal ones, are about 8–10 mm. Among these, stents with low axial force and good biocompatibility are most suitable for clinical treatment [29]. Based on the above references, this study aims to develop a degradable bile duct stent using indirect 3D printing technology. The manufacturing process of the stent is based on the indirect printing of the mold, resulting in the successful printing of a bile duct stent meeting commercial size requirements: an outer diameter of 10 mm and an inner diameter of 8 mm. By employing indirect printing, the stent was degraded, utilizing a cylindrical scaffold with dimensions of 6 mm in diameter and 3 mm in height. The difference in weight loss between the powder of the same material and the cylindrical scaffold shape was observed, providing the basis for optimizing subsequent stent structure designs.

1. In vitro degradation experiments of both powder and stent were conducted, and the degradation of powder and stent materials with different proportions was compared. In the powder degradation experiment, significant weight loss was observed in all four materials, meeting the conditions for material degradation within 4–6 weeks.
2. Human liver cancer cells Hep-3B assessed the material’s biocompatibility and obtained preliminary test results. These served as the basis for the subsequent optimization of the scaffold.

## 2. Methods

LA monomer and GA monomer were purchased from CECHO CHEMICAL, LTD (Toufen, Miaoli, Taiwan). Cell culture media Minimum Essential Medium (MEM), Dulbecco’s Modified Eagle Medium (DMEM), and antibiotics (10,000 IU/mL penicillin and 10,000 μL/mL) were purchased from HyClone™ Laboratories Inc. (San Angelo, TX, USA). Fetal bovine serum (FBS) and Trypsin-EDTA were purchased from Gibco (Carlsbad, CA, USA). Dimethyl sulfoxide (DMSO) and sodium pyruvate were purchased from Sigma-Aldrich (St. Louis, MO, USA). Sodium chloride required for the preparation of phosphate-buffered saline (PBS) was purchased from Amresco (Solon, OH, USA). Potassium phosphate, sodium phosphate, and potassium chloride were purchased from J. T. Baker (Phillipsburg, NJ, USA).

### 2.1. Material Synthesis

Composed of PLA and PLGA in varying ratios (70:30, 50:50, and 30:70), the material was synthesized for follow-up research. The LA and GA monomers were polymerized into a random copolymer using melt polymerization, and the material was synthesized according to the weight percentage distribution ratio, as indicated in Table 1. The process involved the following steps:

1. The GA monomer, LA monomer, and starting group (benzyl alcohol) were weighed and then vacuumed.
2. The catalyst, tin-2-ethylhexanoate (0.0648 mL), was diluted with dry toluene (5 mL), and only 2.5 mL of this solution was used.
3. The catalyst mixture was added to the monomers and the starting group. The toluene was drained after about 10 min, nitrogen (N_2_) was fed into the system, and the mixture was heated to 120–130 °C at 500 rpm to facilitate the reaction, followed by a purification process.
4. For purification, the material was dissolved in dichloromethane (DCM), then crystallized and precipitated using methanol as an auxiliary material. It required maintaining a low temperature for 2–3 days before filtering out the sediment.

The PLA used in the experiments was synthesized using 15 g of lactic acid monomer, and no glycolic acid was used in the formulation. This pure PLA formulation would be used as a control or comparison standard against the various PLGA formulations, which include glycolic acid in different ratios.

The resulting PLGA material, obtained from the melt polymerization of monomers, was produced in powder form. Gel permeation chromatography (GPC) was employed to measure the molecular weight of the synthesized material, using dimethylformamide (DMF) as the solvent and polystyrene (PS) as the standard.

### 2.2. Stent Manufacturing Process

**Figure 1:**
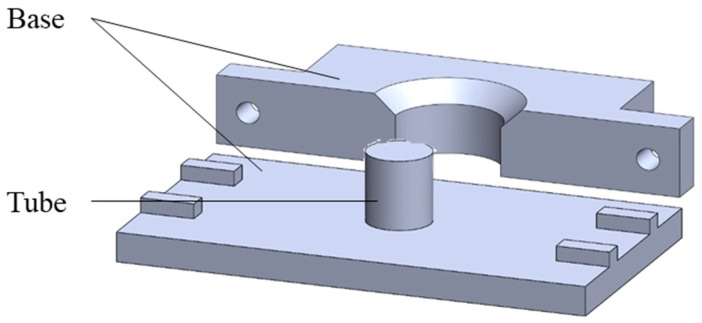
Schematic diagram of the mold.

**Figure 2:**
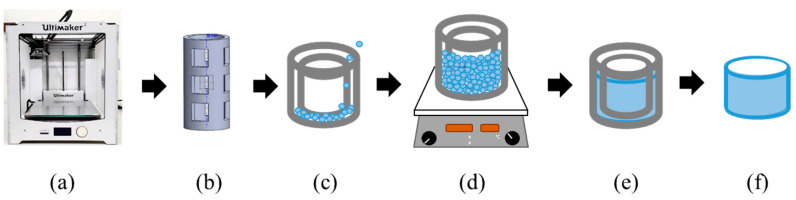
Schematic diagram of indirect printing steps: (**a**) 3D printer, (**b**) mold design and printing, (**c**) molds placed in concentric circles and poured with powder, (**d**) heating the material with a heating stirrer, (**e**) waiting for the fused material to cool, and (**f**) demolding.

This study employed the Ultimaker2 3D printer (3DMart Ltd., New Taipei City, Taiwan) to fabricate stents. The primary printing parameters include nozzle aperture (mm), nozzle temperature (°C), printing platform temperature (°C), and printing speed (%). Adjustments to these parameters were made accordingly. Two Teflon tubes of varying sizes were utilized. The smaller tube had an inner diameter of 6 mm and an outer diameter of 8 mm, while the larger tube had an inner diameter of 10 mm and an outer diameter of 12 mm. These two Teflon tubes were arranged concentrically. PLGA powder was then introduced into the interstitial space between the tubes and heated on the printing platform. Upon heating, the material was compressed to ensure the particles fused firmly; afterward, we stopped the heating. The material was allowed to cool to room temperature. Finally, the stent was extracted using a tool, resulting in an outer diameter of 10 mm, an inner diameter of 8 mm, and a height of 4 mm. The molds include bases and circular tubes of different sizes. The schematic diagram is shown in Figure 1. The molds were combined, PLGA powder was put into the gap between them, and then the material was taken to the heating platform for heating. The material was pressed after heating to make the particles firmly fuse the particles. Then, heating was stopped to cool the material to room temperature, and finally, tools were used to remove the stent to obtain a finished stent. The schematic diagram of the steps is shown in Figure 2.

### 2.3. In Vitro Degradation Experiment

Phosphate-buffered saline (PBS) and PBS supplemented with 10% fetal bovine serum (FBS) were used as degradation solutions, and the samples were placed in a 37 °C incubator. The complete experimental steps of degradation are weighing→disinfection→degradation→cleaning→drying→weighing. We used 75% alcohol to sterilize the sample; it was then dried in the shade and placed under a UV lamp for 15 min. The sample was taken out of the incubator at a predetermined time after degradation, washed three times with sterilized deionized water, and placed in a freeze dryer, where it was dried at 35 °C under vacuum for 3 h, before finally being weighed with an electronic scale.

Each parameter was weighed with three repetitions. The weighing instrument was a Sartorius BP121S precision analytical electronic balance with a minimum reading of 0.1 mg. Each sample was measured three times until the value is stable to obtain the original weight of the sample (*W_i_*). After drying, the electronic balance was used to measure the sample dry weight (*W_t_*); thus, the degradation weight loss percentage can be calculated. Equation (1) is as follows:

(1) $$\mathrm{Weight}\;\mathrm{loss}\;(\%)=\frac{W_{i}-W_{t}}{W_{i}}\times 100\%$$

The percentage weight loss was calculated for triplicate samples and reported in the data as mean ± standard deviation (mean ± SD).

Before and after degradation, the camera and inverted optical microscope (OM) Axiovert 40 CFL were used to observe the change in the appearance of the sample in the degradation solution as the degradation time increased, and the image capture software ZEM lite (ZEM 3.1) was used to capture and analyze the microscopic images.

### 2.4. In Vitro Cytotoxicity Test

To ensure that the pH value of the culture solution soaked in the scaffold is suitable for cell growth, PLA, PLGA (70:30), PLGA (50:50), and PLGA (30:70) were soaked in MEM and DMEM media and measured using acid–base test paper (Macherey-Nagel, 92120, pH-Fix 4.5–10) for four consecutive weeks.

**Figure 3:**
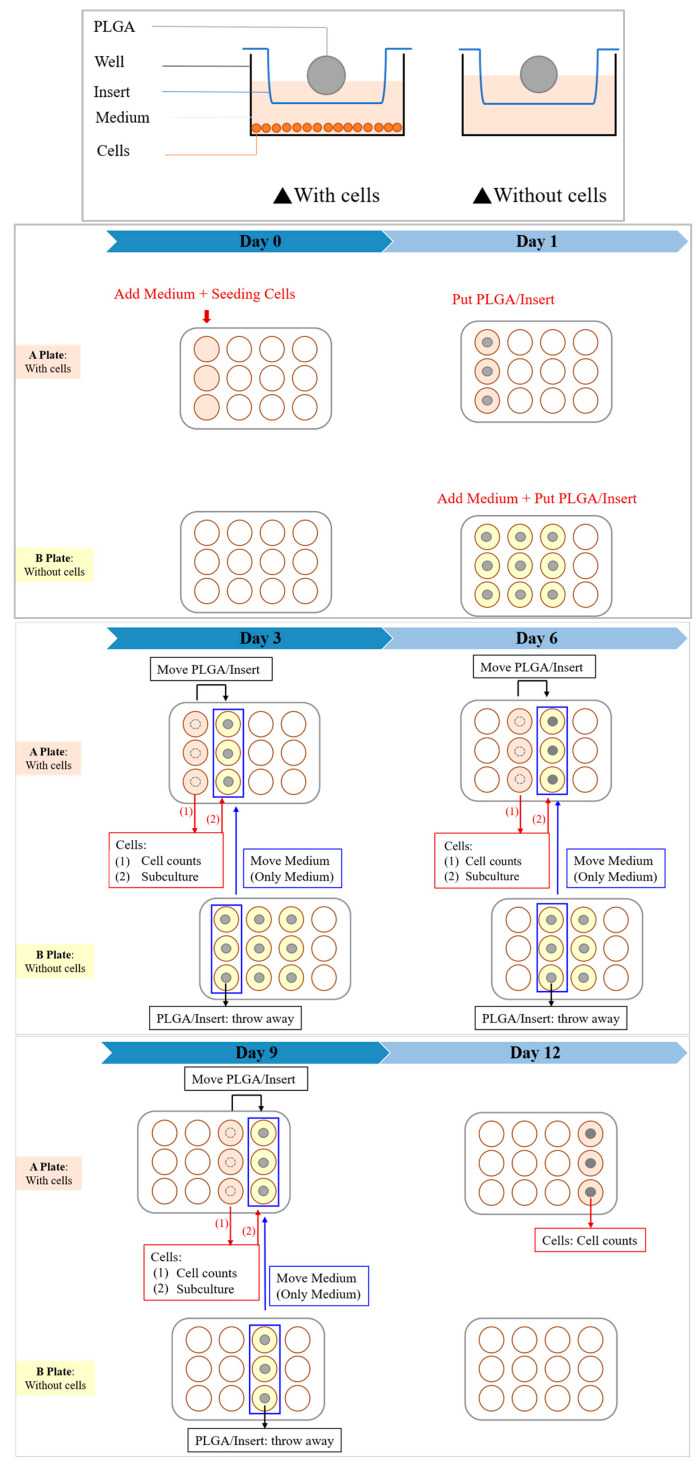
In vitro cytotoxicity test experimental procedure.

Two kinds of cells were used for testing to ensure the authenticity of the toxicity test results: Huh-7 and Hep-3B human liver cancer cells, respectively. Huh-7 uses Dulbecco’s Modified Eagle Medium (DMEM); DMEM is added with 10% FBS and 1% antibiotic. Hep-3B uses Minimum Essential Medium (MEM), supplemented with 10% FBS, 1% antibiotic, and 1% non-essential amino acids (NEAA). The experiment was planned for 12 days, and the cells were seeded in a 12-well culture plate (A plate) on the 0th day at a quantity of 2.5 × 10^4^ cells/well. On the first day (after 16 h), the insert (BD Falcon) and the scaffold were placed in the 12-well plate (A plate), and another 12-well culture plate (B plate) was prepared; the same medium, insert, and scaffold were placed in the B plate as the A plate, but no cells were seeded. On the 3rd, 6th, 9th, and 12th day, the cells in the A plate were subcultures, and the medium soaked in the B plate for the same number of days was used as the replacement medium to achieve the accuracy of the experiment; at the same time, the cell growth and apoptosis patterns were observed with an inverted optical microscope, and the cell survival rate of the cells in the soaked scaffold environment was calculated using an automated cell counter (BIO-RAD, TC-20) and compared with the blank group. The experimental process is shown in Figure 3.

### 2.5. Nano-Indenter Testing System

The instrument used in this experiment was the nano-indenter testing system (Nano indenter XP, Keysight Technologies, Santa Rosa, CA, USA) to test the mechanical properties of materials. The PLGA block produced in the experiment was a composite of LA and GA, used for indentation measurements. Because this material was intended for use as an in-body implant, it must withstand a certain amount of force load; otherwise, its application value is reduced. In nano-indentation tests, the fundamental mechanical properties, such as hardness and Young’s modulus, are determined from the load–displacement curves recorded during the indentation process. Here is a detailed explanation of how these properties are typically extracted:

**Hardness (H)**

1. Load–displacement curve: During the test, a known force (load) is applied to an indenter as it presses into the material’s surface. The displacement of the indenter tip into the material is recorded, creating a load–displacement curve.
2. Maximum load: The curve identifies the maximum force applied during the indentation (*P_MAX_*) and the corresponding displacement.
3. Contact area calculation: The indentation impression’s contact area (A) at maximum load is calculated. This depends on the geometry of the indenter tip (commonly a Berkovich or a spherical tip) and the depth of the indentation.
4. Hardness calculation: Hardness is defined as the material’s resistance to deformation under load. It is calculated using the formula:

$$H=\frac{P_{MAX}}{A}$$

The hardness, in terms of pressure (e.g., GPa), represents the maximum load divided by the contact area at maximum load.

**Young’s Modulus (E)**

Unloading stiffness: The slope of the initial part of the unloading curve (as the indenter is withdrawn from the material) is used to calculate the stiffness (S), the most linear portion of the unloading curve.

Area function: The exact shape and size of the contact area also affect the calculation. For many indenters, an area function based on the depth of penetration describes this relationship.

Young’s modulus calculation: Young’s modulus, which measures the stiffness of the elastic material, is calculated using the unloading stiffness and the contact area. The calculation involves the indenter’s shape and the contact depth. A simplified form of the formula using the Oliver and Pharr method is as follows:

$$E=\frac{1}{\beta }\sqrt{\frac{\pi }{2}\frac{S}{A}}$$

where *β* is a correction factor for the indenter shape (e.g., 1.034 for a Berkovich tip).

These calculations from the load–displacement curves provide quantitative measures of material mechanical properties at tiny scales, making nano-indentation a powerful tool for characterizing materials in research and quality control.

## 3. Results

### 3.1. Material Synthesis

The LA and GA monomers are mixed according to the weight ratio to form PLGA random copolymers by melt polymerization. The molecular weights of the four synthetic materials were measured by gel permeation chromatography (GPC), and the measured data and appearance of the materials are shown in Table 2.

### 3.2. Stent Manufacturing Process

**Figure 4:**
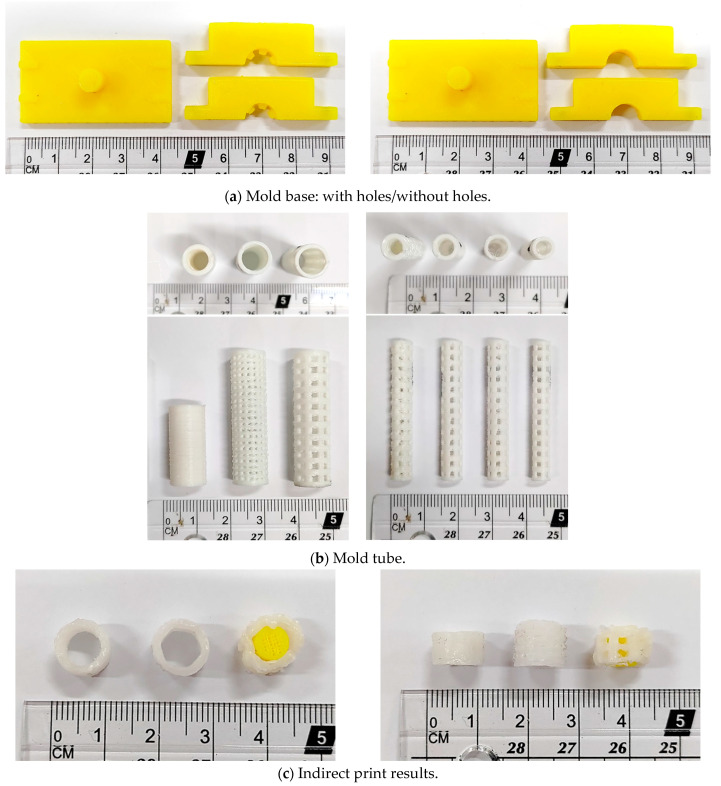
Production results of mold printing support: (**a**) mold base, (**b**) mold tube, and (**c**) finished product.

A 3D printer can be used to manufacture molds with and without holes, as shown in Figure 4a, and round tubes of different sizes can be produced, as shown in Figure 4b. Various brackets can be made using molds, as shown in Figure 4c; from left to right are brackets without holes, brackets with threads, and brackets with holes. Due to the limitation of the manufacturing process, only the mold without holes is used for indirect printing. 

The process temperature and test results of the four materials are shown in Table 3. The manufacturing temperature ranges of PLA, PLGA (70:30), PLGA (50:50), and PLGA (30:70) are 60–80 °C, 100–150 °C, 70–90 °C, and 220–250 °C, respectively. It can be found that the ratio between the manufacturing temperature and the material is not linear: among PLGA (70:30), PLGA (50:50), and PLGA (30:70), the process temperature of PLGA (50:50) is the lowest, which is the same result as the fastest degradation rate of PLGA (50:50) presented in the literature. 

**Figure 5:**
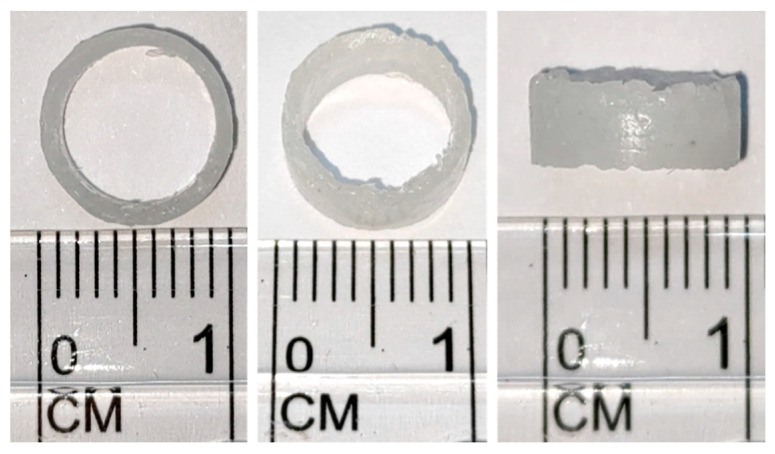
Indirect printing of finished product drawing of final size.

After determining the manufacturing temperature, indirect printing is performed with the mold to produce a standard bracket with an outer diameter of 10 mm, an inner diameter of 8 mm, and a height of 4 mm, as shown in Figure 5.

**Figure 6:**
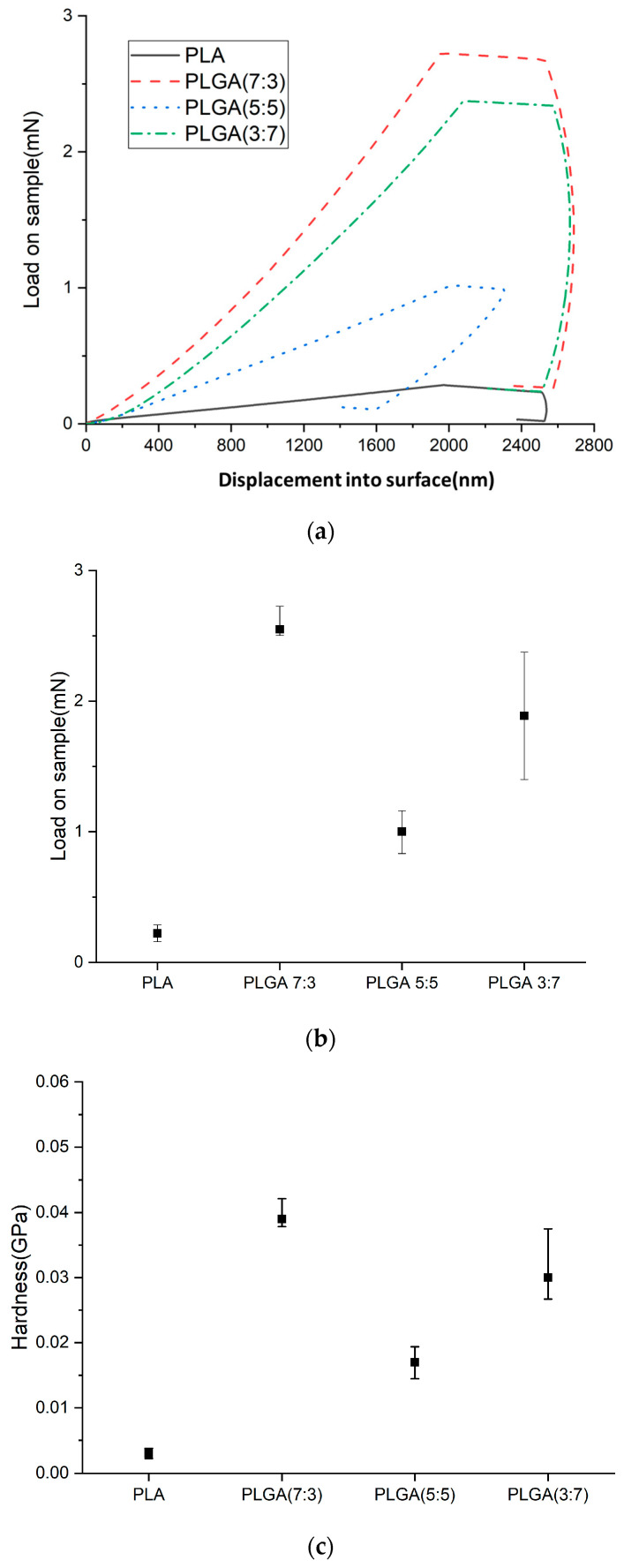

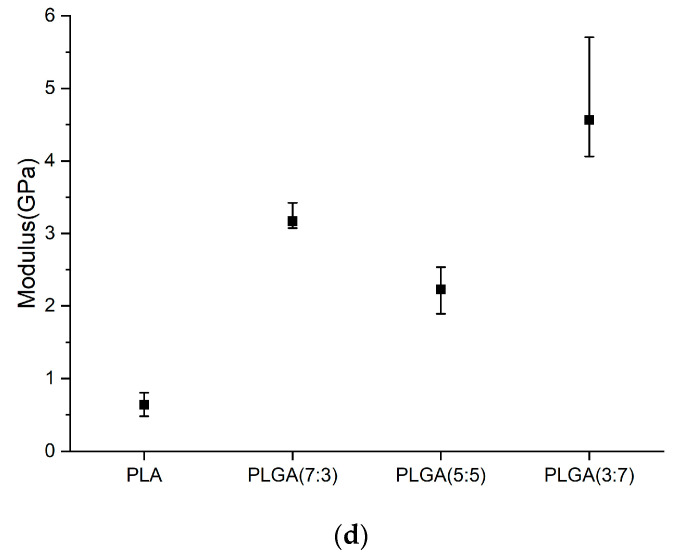
(**a**) Load and unload curve diagrams, (**b**) loads, (**c**) hardness, and (**d**) Young’s modulus of PLA and PLGA composite materials (70:30, 50:50, and 30:70).

Figure 6a–d show load and unload curve diagrams, loads, hardness, and Young’s modulus of PLA and PLGA composite materials (70:30, 50:50, and 30:70).

When the composition ratio of a material changes, its mechanical properties could exhibit non-linear responses. For example, chemical bonds or intermolecular forces between different components could affect the material’s elasticity and tensile strength. Weak interactions, such as hydrogen bonds or van der Waals forces, may increase toughness but reduce hardness. Additionally, changes in microstructure could also be a factor influencing these properties. Since this material is intended for an in-body stent, it must withstand a specific force load. Therefore, it is essential to understand the characteristics of PLGA material, such as the effect of adding GA to PLA on the mechanical properties of the composite material PLGA (e.g., Young’s modulus and load and unload curves).

The material determined the load capacity in indentation testing and compared the degradation rate in degradation experiments. Young’s modulus and hardness could be used as a reference if the material was expected to maintain the load capacity when subjected to degradation tests. Young’s modulus and hardness of PLA were found to be approximately 0.639 GPa and 0.004 GPa, respectively. After adding three different weight percentages of GA, the PLGA composites showed higher Young’s modulus and hardness values than pure PLA, as shown in Table 4. The maximum values for Young’s modulus and hardness were increased to 3.169 GPa and 0.039 GPa, respectively. Among them, the Young’s modulus and hardness of PLGA (5:5) (compared to PLGA (7:3) and PLGA (3:7)) are relatively low, at 0.544 GPa and 0.017 GPa, respectively, which may be related to the degradation rate.

The nano-indenter system was used to measure load–unload curves of materials such as PLA and various PLGA composites. Local behavior pertained to the material’s response to forces and deformations at the micro- or nano-scale, including local hardness, elastic modulus, and compressive resistance, which were crucial for evaluating the material’s initial mechanical performance and biocompatibility. Global behavior encompassed the material’s performance under forces and environmental conditions over longer durations. For example, while nano-indentation tests may indicate high hardness and elastic modulus, the material may exhibit different behaviors under cyclic loading or in various pH conditions encountered within the human body.

### 3.3. In Vitro Degradation Experiment

**Figure 7:**
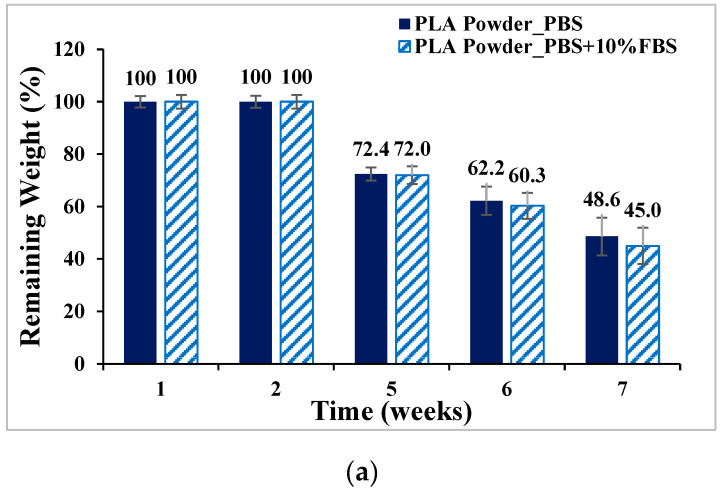

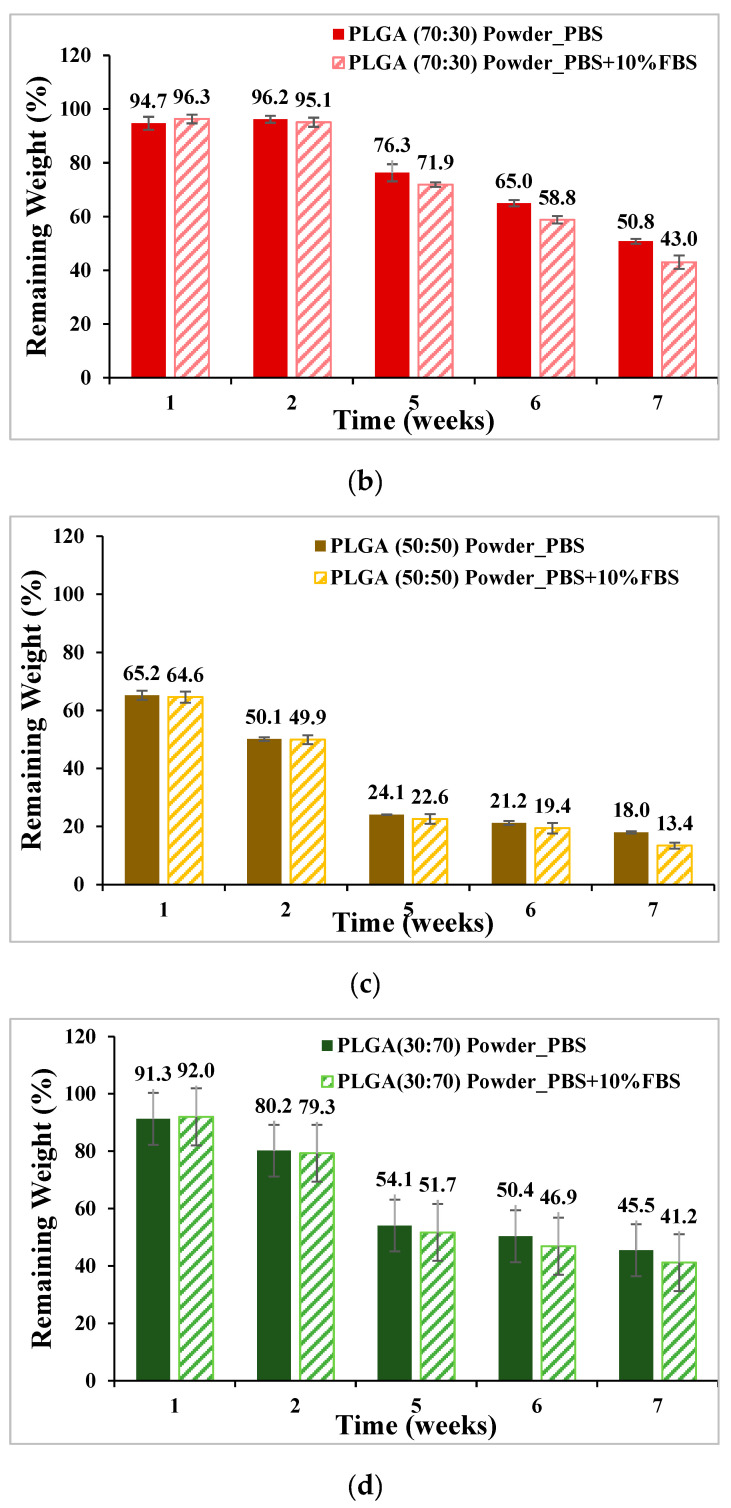
Weight loss curve of synthetic material powder degradation for seven consecutive weeks (mean ± SD): (**a**) PLA, (**b**) PLGA (70:30), (**c**) PLGA (50:50), and (**d**) PLGA (30:70).

PLA, PLGA (70:30), PLGA (50:50), and PLGA (30:70) powders were degraded in PBS and PBS degradation solutions with 10% FBS, respectively, and the weight loss percentages are shown in Figure 7. The ratio of sample to degradation solution is 1:25. The weight of the four materials all decreased with the increase in the degradation cycle, among which PLGA (50:50) lost the most weight; by week 7, the weight had decreased from 100% to 13.4% by soaking in PBS degradation solution containing 10% FBS.

**Figure 8:**
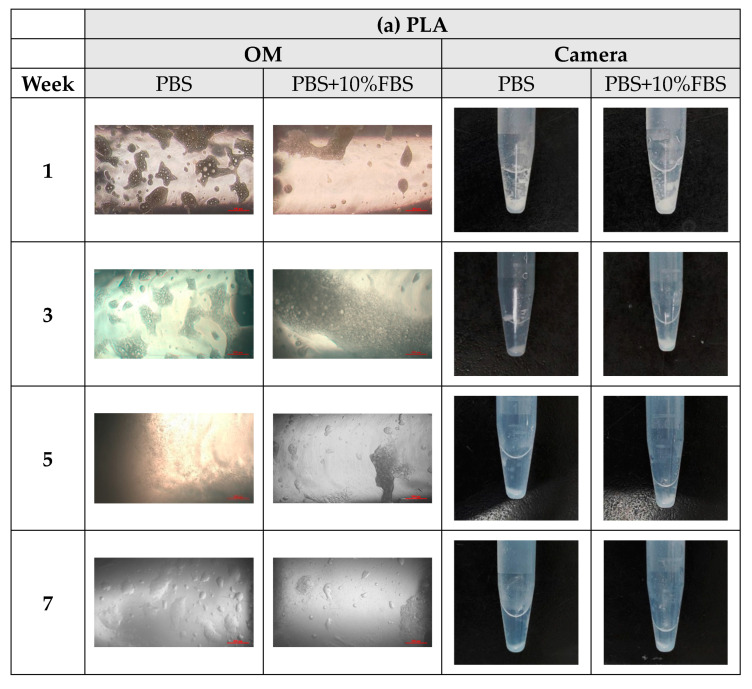

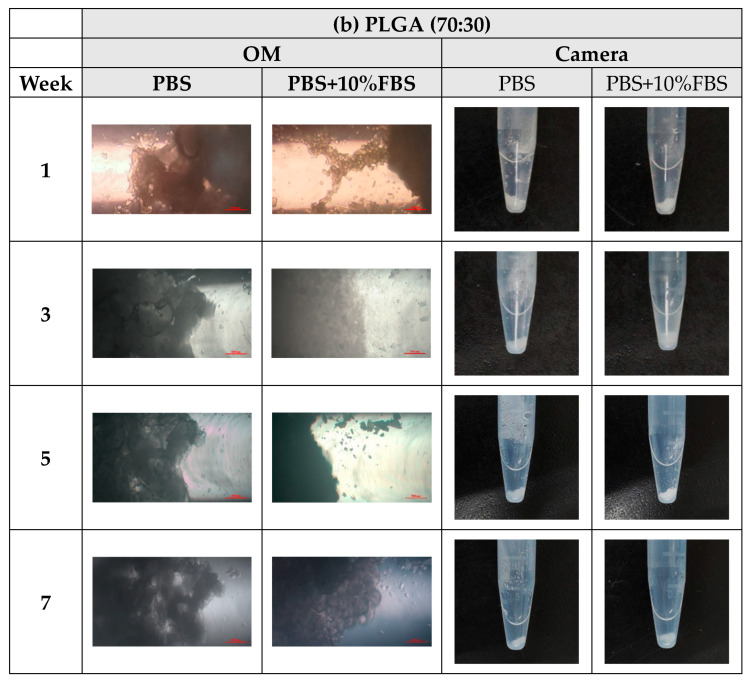

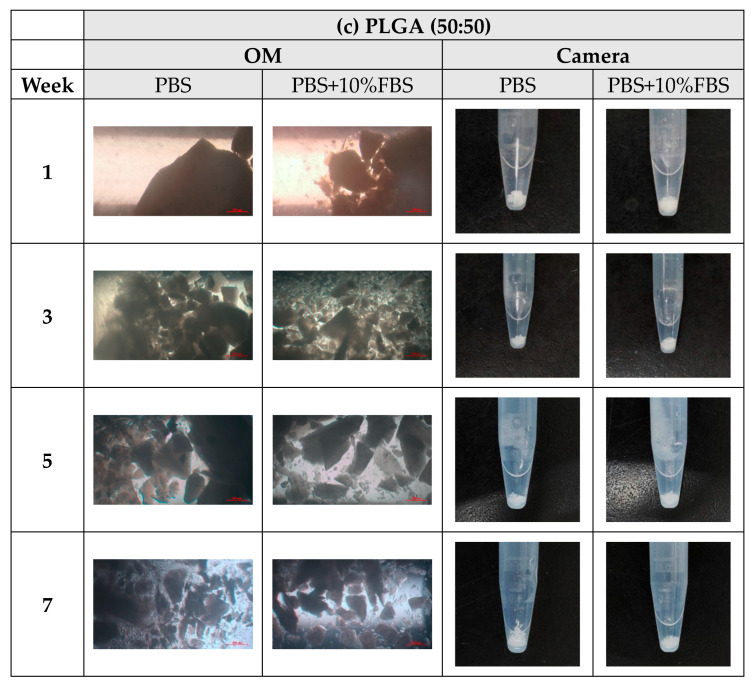

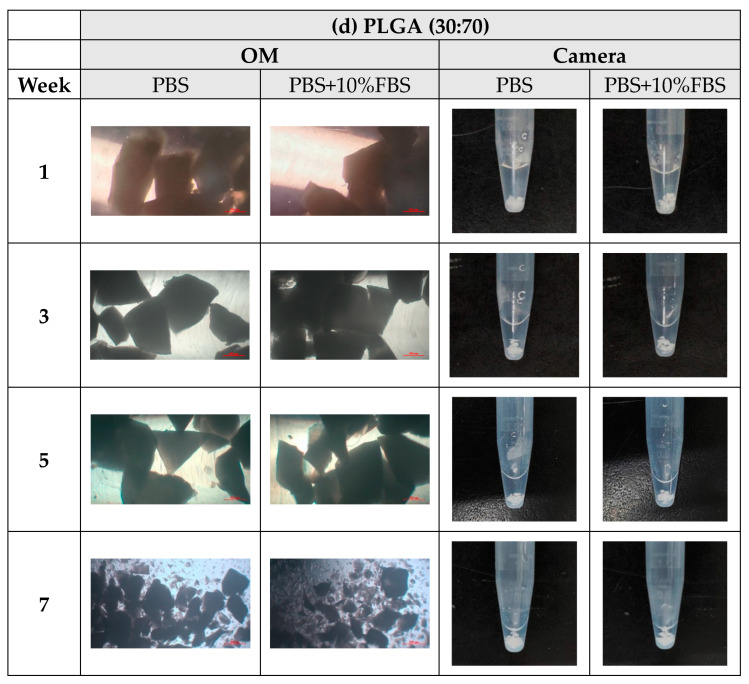
The microscopic changes of the powder degraded under the inverted optical microscope and the naked eye (camera) for 7 weeks (scale bar = 500 μm): (**a**) PLA, (**b**) PLGA (70:30), (**c**) PLGA (50:50), and (**d**) PLGA (30:70).

During the degradation experiments of PLA, PLGA (70:30), PLGA (50:50), and PLGA (30:70) powders, the appearance changed under the inverted optical microscope and the naked eye (photographed by the camera), as shown in Figure 8a–d. It can be observed that the transparency of the four material powders gradually changes from opaque to transparent, and the appearance gradually degrades from larger particles to smaller powders, indicating that the degradation rate is fast and consistent with the results of the weight loss curve.

**Figure 9:**
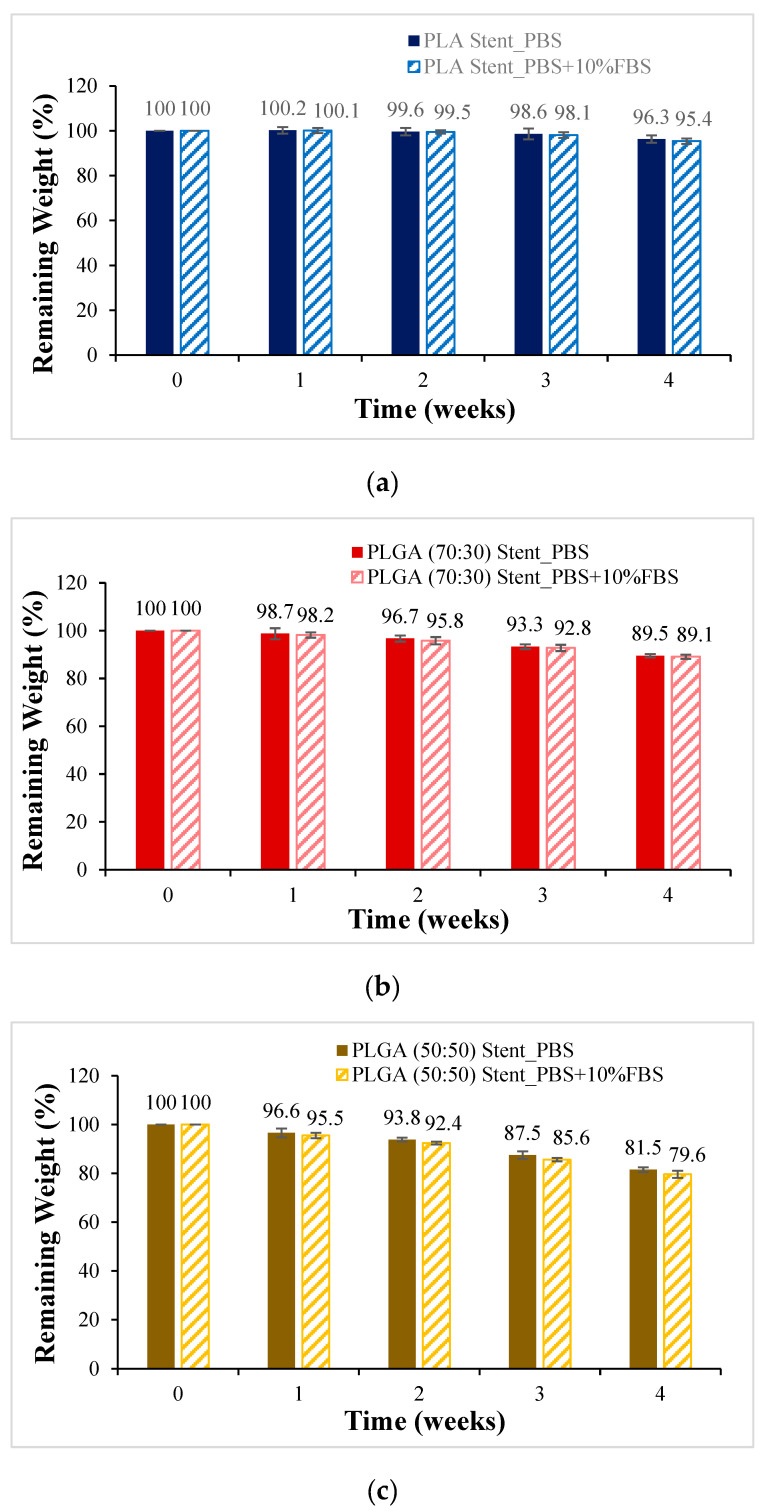

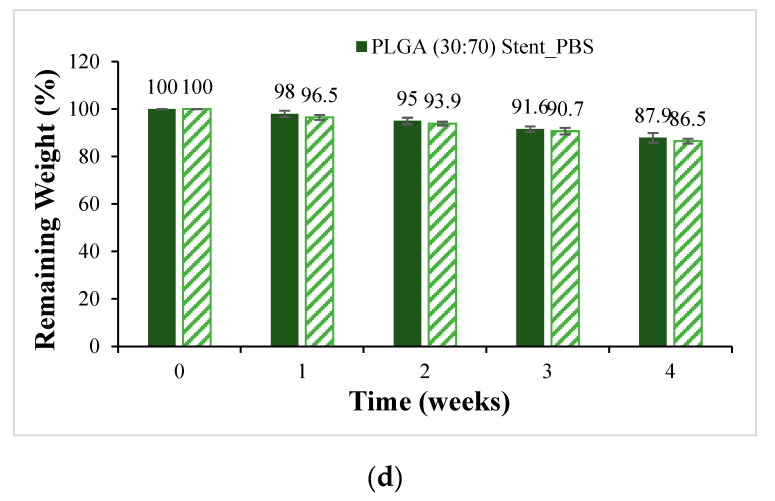
Weight loss curves of synthetic scaffolds at four weeks of degradation (mean ± SD): (**a**) PLA, (**b**) PLGA (70:30), (**c**) PLGA (50:50), and (**d**) PLGA (30:70).

PLA, PLGA (70:30), PLGA (50:50), and PLGA (30:70) materials are made into a tubular scaffold; to minimize the appearance error of the sample, the sample for the degradation experiment was made into a cylindrical bracket with a diameter of 6 mm and a height of 3 mm. It was degraded in PBS and PBS degradation solution with 10% FBS added, respectively, and the weight loss percentage is shown in Figure 9. The ratio of sample to degradation solution is 1:25. The material with the most significant change in weight loss is still PLGA (50:50). After four weeks of degradation, the sample weight dropped from 100% to about 80%. Compared with powder degradation, the weight loss is less obvious, which can be attributed to the lower degradation rate due to the absence of pores in the scaffold.

**Figure 10:**
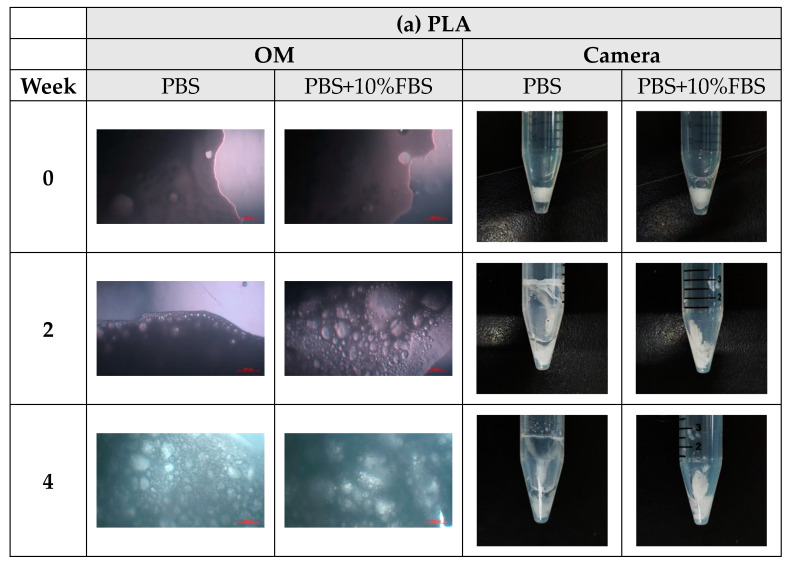

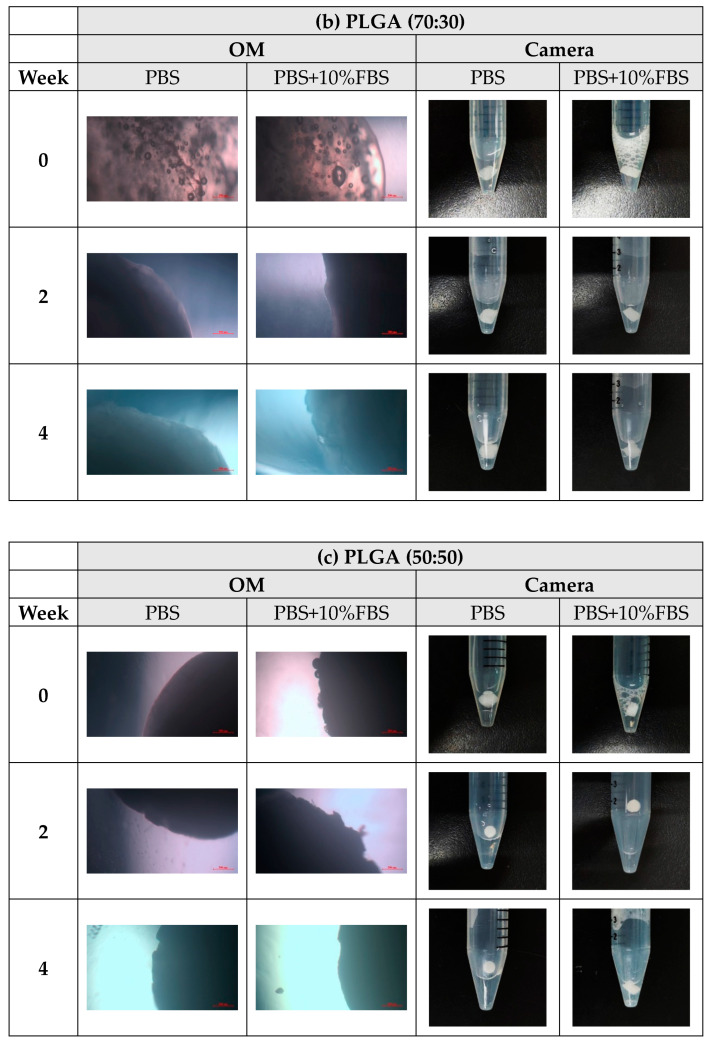

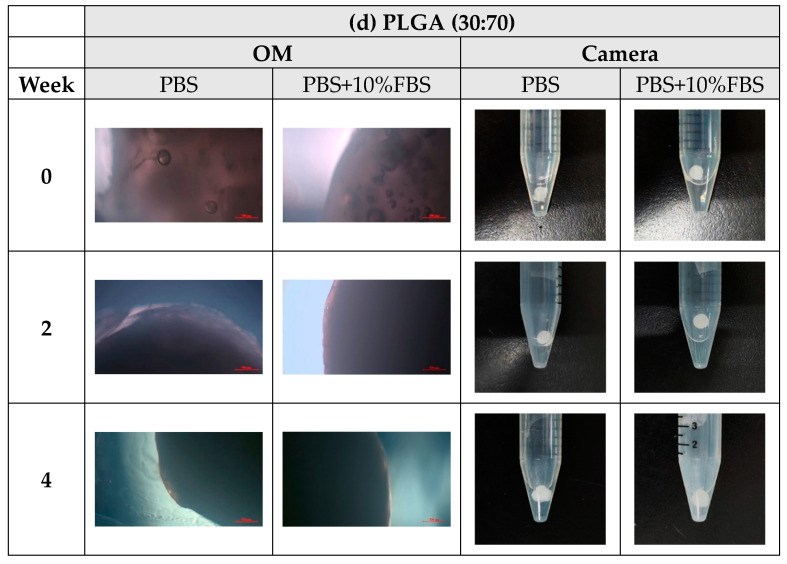
The microscopic changes of the stent degraded under the inverted optical microscope and the naked eye (camera) for 7 weeks (scale bar = 500 μm): (**a**) PLA, (**b**) PLGA (70:30), (**c**) PLGA (50:50), and (**d**) PLGA (30:70).

During the degradation process of the scaffolds of PLA, PLGA (70:30), PLGA (50:50), and PLGA (30:70), the appearance changed under the inverted optical microscope and the naked eye (photographed by the camera), as shown in Figure 10a–d. The transparency and size of the scaffold appearance of PLGA (70:30), PLGA (50:50), and PLGA (30:70) did not change much during the four-week degradation period, and the scaffold appearance hardly changed. Only the PLA stent changed significantly: the appearance could not maintain the prototype after one week of degradation, and the transparency of the stent also changed from opaque to transparent.

### 3.4. In Vitro Cytotoxicity Test

**Figure 11:**
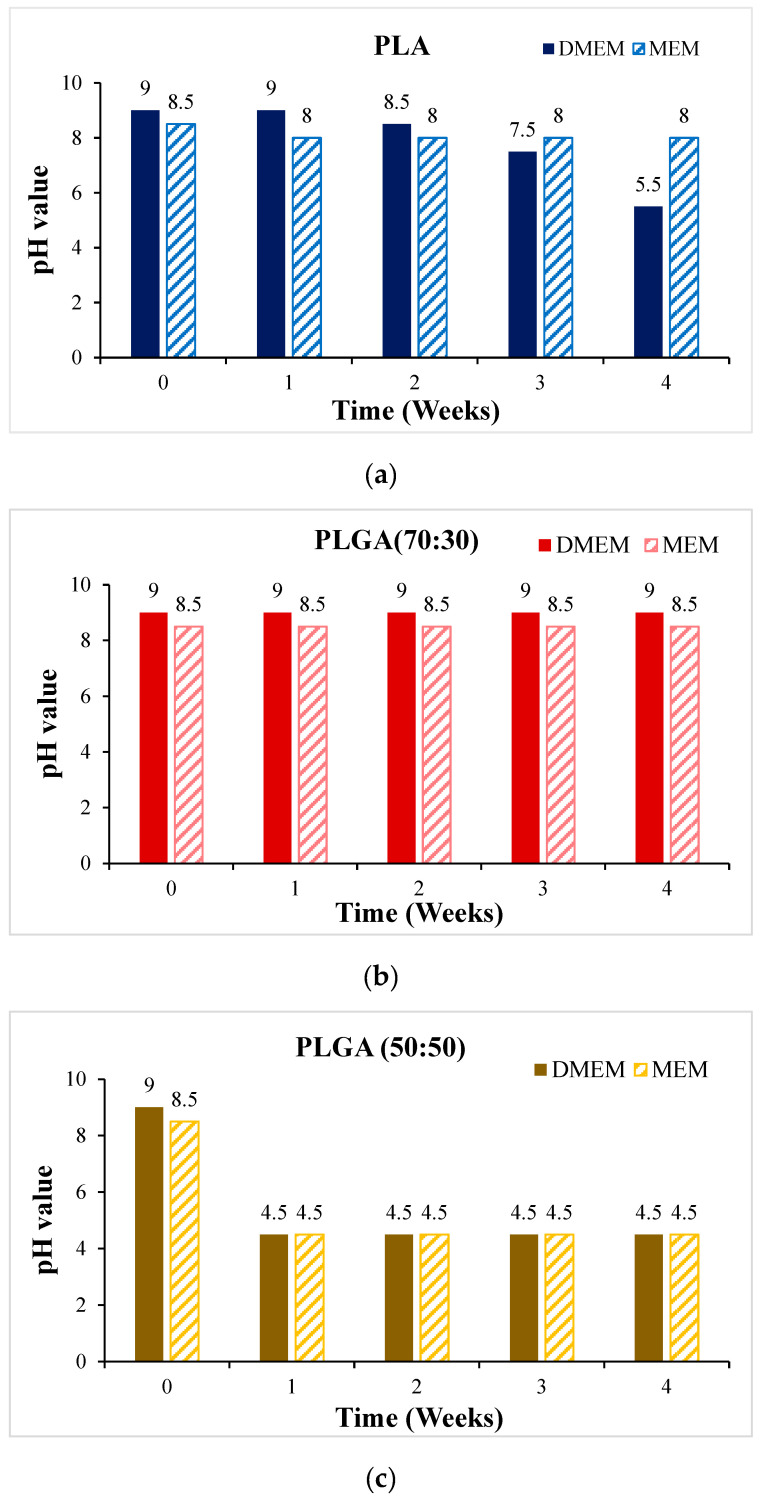

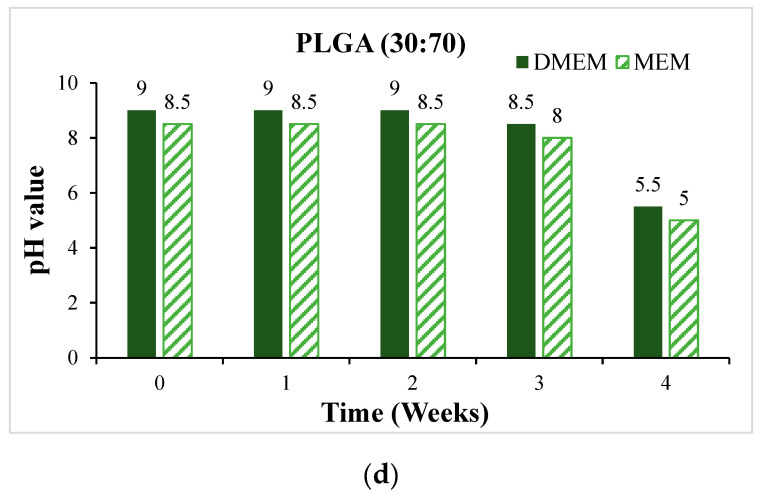
The change of pH value of the scaffold soaked in DMEM and MEM culture solution for 4 weeks: (**a**) PLA, (**b**) PLGA (70:30), (**c**) PLGA (50:50), and (**d**) PLGA (30:70).

**Figure 12:**
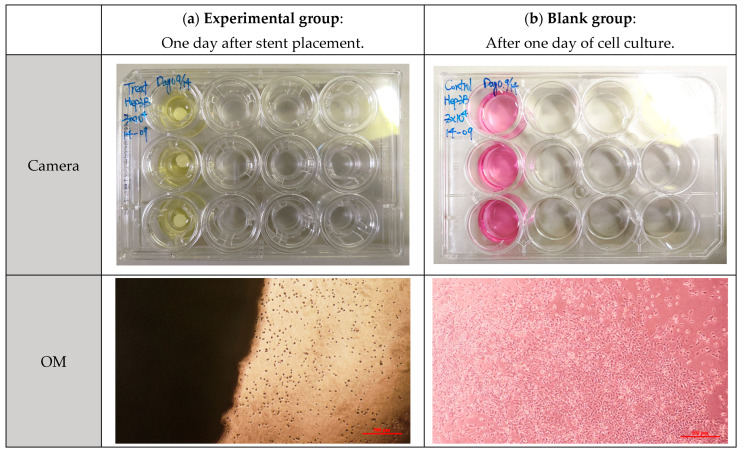
Cytotoxicity test of PLGA (50:50) scaffold in Hep3B cell culture solution: (**a**) experimental group and (**b**) blank group.

Before the cytotoxicity test, the material was soaked in DMEM and MEM culture solution, respectively, and the change in pH value with soaking time was measured, as shown in Figure 11. It can be seen that the pH values of PLA, PLGA (50:50), and PLGA (30:70) all decreased significantly during soaking. To ensure that the cells can grow and develop in the most suitable environment and ensure the reliability of in vitro cytotoxicity tests and the success of subsequent scaffold implantation, we need to optimize and improve the process of synthetic materials for the problem of over-acidic pH. By adjusting the manufacturing process of synthetic materials, the pH value of the material can be effectively controlled to ensure that it meets the standards in vivo. PLGA (50:50), which has a significant degradation rate, was selected for the cytotoxicity test, and Hep3B cells were randomly selected as test objects: if the sample is soaked in a solution containing Hep3B cells, the cells can maintain a growth state for some time. As shown in Figure 12, it is preliminarily judged that the degradation rate of PLGA (50:50) is the fastest. The magnification of the OM is 40×, and the scale bar in the images represents 500 μm. Figure 12a shows the experimental group, where cell apoptosis can be observed, while Figure 12b presents the blank group, where cells continue to grow. Hence, the concentration of lactic acid and glycine in the solution is higher, which promotes a rapid drop in pH value and causes cell apoptosis. It can be seen that the bile duct stent gradually degrades over time.

## 4. Conclusions

Biodegradable bile duct stents were synthesized and characterized from a blend of PLA and PLGA using indirect 3D printing technology in this study. The optimization of PLA and PLGA ratios, specifically the PLGA (50:50) composition, demonstrated the fastest degradation rate, achieving a significant weight reduction to 13.4% over four weeks, suitable for applications requiring short-term implantation such as temporary stent placement. In vitro cytotoxicity tests were also conducted. This study used human liver cancer cells (Hep3B) to assess biocompatibility, where the cells were able to survive for an extended period. The experiments validated the material’s ability to maintain physical integrity and induce minimal cytotoxic effects in in vitro settings, highlighting its potential for short-term therapeutic applications in managing bile duct obstructions. This innovation not only aligns with clinical needs but also offers a promising avenue for future enhancements in polymer-based, biodegradable medical devices.

## Figures and Tables

**Table 1:** Weight formulation of PLGA melt polymerization.

	LA Monomer	GA Monomer
PLA	15 g	--
PLGA (70:30)	10.5 g	4.5 g
PLGA (50:50)	7.5 g	7.5 g
PLGA (30:70)	4.5 g	10.5 g

**Table 2:** Properties of PLGA materials synthesized by melt polymerization.

Material	PLA	PLGA (70:30)	PLGA (50:50)	PLGA (30:70)
M_n_ (g/mole)	4000	16,000	16,000	16,000
Appearance	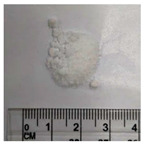	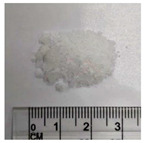	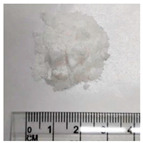	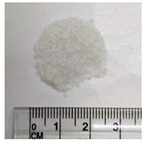

**Table 3:** Material process temperature test results.

Material	PLA	PLGA (70:30)	PLGA (50:50)	PLGA (30:70)
Temperature	60–80 °C	100–150 °C	70–90 °C	220–250 °C
Test record	60 °C 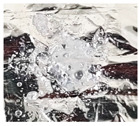	100 °C 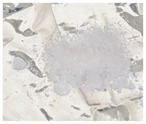	70 °C 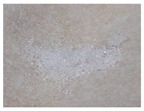	220 °C 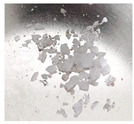
	70 °C 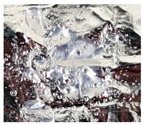	120 °C 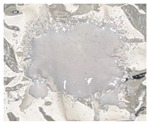	80 °C 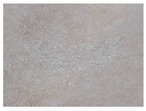	250 °C 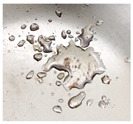
	80 °C 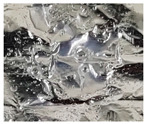	150 °C 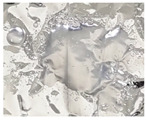	90 °C 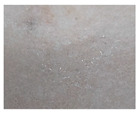	--

**Table 4:** Young’s modulus and hardness.

	Young’s Modulus (GPa)	Hardness (GPa)
PLA	$0.639_{-0.160}^{+0.166}$	$0.003_{-0.00075}^{+0.00078}$
PLGA (7:3)	$3.169_{-0.095}^{+0.253}$	$0.039_{-0.00117}^{+0.00312}$
PLGA (5:5)	$2.226_{-0.334}^{+0.312}$	$0.017_{-0.00255}^{+0.00238}$
PLGA (3:7)	$4.564_{-0.502}^{+1.141}$	$0.03_{-0.0033}^{+0.0075}$

## Data Availability

The data presented in this study are available on request from the corresponding author.
